# Voltage-gated potassium channels are involved in oxymatrine-regulated islet function in rat islet β cells and INS-1 cells

**DOI:** 10.22038/ijbms.2021.52449.11850

**Published:** 2021-04

**Authors:** Jingying Gao, Lixia Xia, Yuanyuan Wei

**Affiliations:** 1 Department of Pediatrics, Shanxi Medical University, Taiyuan, China; 2 Pediatric internal Medicine, Children’s Hospital of Shanxi Province, Shanxi Medical University, Taiyuan, China

**Keywords:** Apoptosis, Diabetes mellitus, Insulin secretion, Oxymatrine, Potassium channel, Voltage-gated

## Abstract

**Objective(s):**

Oxymatrine can regulate glucose metabolism. But the underlying mechanisms remain unclear. We investigated the relationship of oxymatrine and voltage-gated potassium (Kv) channel in rat islet β cells and INS-1 cells.

**Materials and Methods:**

Insulin secretion and Kv channel currents were tested by radioimmunoassay and patch-clamp technique, respectively. The INS-1 cell viability was detected using cell counting kit-8 experiments. Flowcytometry analysis and western blot were employed for cell apoptosis and protein levels, respectively. INS-1 cell proliferation was assessed by the 5-Ethynyl-2’- deoxyuridine method.

**Results:**

Oxymatrine potentiated insulin secretion at high glucose (*P*<0.01 vs 11.1 G, *P*<0.01 vs 16.7 G) and inhibited KV currents at 40 mV (45.73±15.34 pA/pF for oxymatrine, 73.80±19.23 pA/pF for control, *P*<0.05). After the INS-1 cells were treated with oxymatrine for 12 and 24 hr, KV2.1 channel protein was up-regulated (*P*<0.01 vs Control). At the same time, compared with the high glucose and high fat group, cell viability and proliferation ability were increased (*P*<0.01). The cell apoptotic rate was reduced, reaching 17.30%±1.00% at 12 hr and 10.35%±1.52% at 24 hr (*P*<0.01). These protective effects of oxymatrine were reversed by using Stromatoxin-1, a kv channel inhibitor.

**Conclusion:**

The results indicate that oxymatrine can stimulate insulin secretion and decrease kv channel currents in islet β cells. Besides, oxymatrine also increases cell viability, proliferation, and reduces cell apoptosis in INS-1 cells. The effects of oxymatrine are related to kv channels. This finding provides new insight into the mechanisms of oxymatrine-regulated islet function.

## Introduction

Diabetes mellitus is a chronic progressive disease with high incidence. It is characterized by insufficient insulin secretion and/or cell dysfunction. It can lead to disability and death. According to a report by the International Diabetes Federation, the number of people with diabetes is on the rise in 2019 worldwide. The average growth rate is 51% (1). In diabetes, type 2 diabetes mellitus (T2DM) accounts for 90% (2). The etiology and pathogenesis are not fully understood. The basic pathophysiology is insulin resistance and pancreatic β cell hypofunction. How to control blood glucose, promote β cell proliferation, and inhibit β cell apoptosis is an important basis for treatments of T2DM.

Depending on the biophysical properties, potassium (K^+^) channels are classified into different families and subfamilies (3). Delayed rectifiers, such as Kv1.2, Kv2.1, and Kv3.1, have higher activation thresholds and exhibit very slow inactivation (4, 5)**. **In rat islet β cells, glucose increases the intracellular ATP levels. The resultant closure of ATP-sensitive potassium (K_ATP_) channels causes cell membrane depolarization and activates voltage-gated calcium (Ca_V_) channels. Subsequently, Ca^2+^ enters the cells and insulin secretion is promoted (6-8). Meanwhile, membrane depolarization also activates the voltage-gated potassium (Kv) channels. The outflow of K^+^ leads to cell membrane repolarization, which prevents Ca^2+^ entry through voltage-gated calcium channels and inhibits insulin secretion (7, 9, 10). 

In rodents, many studies have indicated that the repolarization of pancreatic β cell action potentials is largely mediated by Kv2.1 (11). For example, a study demonstrated that adenovirus-mediated expression of a C-terminal truncated Kv2.1 subunit, specifically eliminating Kv2 family currents, inhibits delayed rectifier currents by 60–70% in rat islets cells. Glucose-stimulated insulin secretion from rat islets is increased by 60% (12). In Kv2.1^-/-^ C57BL/6 mice, glucose-stimulated action potentials recorded by the perforated patch-clamp show a significantly prolonged duration compared to control islets at high glucose concentration (14.4 mmol/l) (11). 

Kv2.1 is also involved in the apoptosis processes of neuron and pancreatic β cells (13-15). Kv channels participate in staurosporine or thapsigargin-induced β cell apoptosis. Overexpression of Kv2.1 in INS 832/13 cells potentiate apoptosis in response to mitochondrial and endoplasmic reticulum stress (13). SP6616 (a Kv2.1 inhibitor) suppresses the streptozocin-induced INS 832/13 cell apoptosis in MTT assay. In addition, Western blot experiments show that SP6616 inhibits the streptozocin-induced increases in both the protein level of cleaved caspase 3 and the activity of caspase 3/7 (15). 

Oxymatrine, the main component of a traditional Chinese herb, *Sophora flavescens* Ait, has anti-inflammatory, antiviral, liver-protecting, anti-tumor, immunomodulatory, and antioxidant effects (16-19). At present, oxymatrine has been used clinically in China, mainly for the treatment of liver diseases (20, 21). In recent years, some studies have found that oxymatrine can regulate glucose metabolism. Oxymatrine significantly increases the number of pancreatic islets, promotes insulin secretion, and improves insulin sensitivity in diabetic rats (22). After streptozotocin-induced diabetic rats were intragastrically administered oxymatrine for 7 weeks, the blood glucose levels were decreased and insulin levels were increased in a dose-dependent manner (23). Furthermore, oxymatrine has been shown to have anti-apoptotic effects. By reducing the caspase-3 level and increasing Bcl-2/Bax level, oxymatrine can suppress neuronal apoptosis, improve learning function and alleviate cognitive impairment in diabetic rats (24, 25). Oxymatrine also down-regulates the apoptosis of hepatocytes in rats with acute liver injury by inhibiting the TLR4/PI3K/Akt/GSK-3β pathway (26). It has been indicated that oxymatrine can prevent cardiomyocyte apoptosis and improve the cardiotoxicity induced by doxorubicin and aldosterone in rats (27, 28). 

Some studies have proved that oxymatrine can regulate the K^+^ channel. Oxymatrine decreases the peak amplitudes of delayed rectifier K^+^ currents in clonal pheochromocytoma (PC12) cells (29). In HEK293 cells that stably expressed the wild-type human ether-a-go-go-related gene (hERG), oxymatrine inhibits hERG tail current in a concentration-dependent manner (30). But, the role of K_V_ channels in oxymatrine-regulated islet cell function remains unclear. Therefore, we investigated the relationship of K_V_ channels and oxymatrine in rat islet β cells and rat insulinoma (INS-1) cells. The results demonstrate that oxymatrine increases insulin secretion, inhibits INS-1 cell apoptosis, promotes INS-1 cell viability and proliferation, which relates to the dual effects of oxymatrine on K_V_ channels.

## Materials and Methods

***Animals and cell line***

Male Sprague-Dawley (SD) rats (250 ± 20 g) were supplied by the Animal Experimental Center of Shanxi Medical University (Taiyuan, China). All rats were housed in air-conditioned animal cages (approximately 25 ± 2 °C, 55–60% humidity, 12 hr of light-dark cycles). They could get food and water freely. The animals were carefully treated in accordance with ethical guidelines for Animal Use of Shanxi Medical University (Taiyuan, China). The INS-1 cell line was purchased from Shanghai AiYan Biological Technology Co., LTD (Shanghai, China).

***Islet isolation and cell culture***

As described in the previous experimental procedure (18, 19), pancreatic islets were acquired from SD rats by Collagenase P (1 mg/ml, Roche, Indianapolis, USA) digestion and histopaque-1077 (Sigma-Aldrich, USA) density gradient separation. After digestion for 5 min by Dispase II (Roche, Indianapolis, USA), the isolated islets were dispersed to single islet cells. Pancreatic islets and islet cells were cultured in RPMI 1640 medium (Gibco, Grand Island, NY, USA) containing glucose (11.1 mmol/l, Sangon Biotech Co. LTD., Shanghai, China), fetal bovine serum (10%, Gibco, Grand Island, NY, USA), penicillin (100 U/ml, Sigma-Aldrich, St. Louis, MO), and streptomycin (100 μg/ml, Sigma-Aldrich, St. Louis, MO) at 37 °C in 5% CO_2_ humidified atmosphere. The INS-1 cells were maintained in RPMI-1640 medium supplemented with penicillin (100 U/ml), streptomycin (100 μg/ml), sodium pyruvate (0.11 g/l, Sangon Biotech Co. LTD., Shanghai, China), β-mercaptoethanol (50 μmol/l, Gibco, Grand Island, NY, USA) and fetal bovine serum (10%) at 37 °C in 95% air with 5% CO_2_. 

***Insulin secretion experiments***

The islets were pre-incubated with Krebs-Ringer bicarbonate-HEPES (KRBH) buffer containing 2.8 mmol/l glucose for 0.5 hr and the supernatant was discarded. Subsequently, the islets were incubated with KRBH buffer including 2.8 mmol/l, 11.1 mmol/l, or 16.7 mmol/l glucose in the presence or absence of oxymatrine (Sigma-Aldrich, St. Louis, MO) for 0.5 hr. The supernatant was collected and tested for insulin concentration by an Iodine [^125^I] Insulin Radioimmunoassay Kit (Beijing North Institute of Biological Technology, China). The islets in each tube were lysed with 70% acid-ethanol solution (Ethanol/water/HCl (vol/vol) 150:47:3) and measured for insulin content. A composition of Krebs-Ringer bicarbonate-HEPES (KRBH) buffer was as follows(mmol/l):128.8 NaCl;1.2 KH_2_PO_4_;4.8 KCl; 2.5 CaCl_2_·2 H_2_O; 1.2 MgSO_4_; 10 HEPES; 5 NaHCO_3_ and 2% bovine serum albumin (Solarboi, Beijing, China) at pH 7.4.

***Patch-clamp experiments***

In order to record voltage-gated potassium (K_V_) channel currents, the solution was prepared. The intracellular solution included (mmol/l): 10 NaCl; 140 KCl; 10 HEPES; 0.05 EGTA; 1 MgCl_2_, pH 7.3 adjusted with KOH. The extracellular solution contained (mmol/l):141.9 NaCl; 5.6 KCl; 1.2 MgCl_2_; 11.1 glucose; 5 HEPES, pH 7.4 with NaOH. The electrodes were pulled by Narishige MODEL PP-830 micropipette puller (Narishige Co., Tokyo, Japan) and polished to resistances of 4 to 7 MΩ by MICRO FORGE MF-200 (World Precision Instruments Inc., USA). K_V_ channel currents were recorded in whole-cell voltage-clamp mode with EPC-10 amplifier and PULSE software (HEKA Electronik, Lambrecht, Germany) from a holding potential of -70 mV to various test pulses (-70 to 80 mV) in 10 mV steps. Islet β cells were recognized by their membrane capacitance [>7 picofarads (pF)] (31).

***CCK-8 assay***

Cell counting kit-8 (CCK-8, Beyotime Biotechnology, Shanghai, China) was used to detect the viability of the INS-1 cells. The INS-1 cells were seeded in 96-well plates with a density of 8 × 10^3^ cells / well. The INS-1 cells were divided into 4 groups: control group; high glucose (30 mmol/l glucose) + high fat (400 μmol/l palmitic acid sodium (Sigma-Aldrich, St. Louis, MO)) group; high glucose (30 mmol/l glucose) + high fat (400 μmol/l palmitic acid sodium) + oxymatrine (10 μmol/l) group; high glucose (30 mmol/l glucose) + high fat (400 μmol/l palmitic acid sodium) + oxymatrine (10 μmol/l) + stromatoxin-1 (a K_V_ channel inhibitor, 100 nmol/L, MuseChem, New Jersey, USA) group. After the cells were incubated at 37 °C with 5% CO_2_ humidified atmosphere for 12 hr or 24 hr, 10 μl CCK-8 was added to each well for 4 hr and the optical density (OD) at 450 nm was determined by Berthold LB941 multifunctional microporous reader (Berthold Technologies, Germany). 

***Flow cytometry analysis***

For cell apoptosis detection, Annexin V Alexa Fluor™ 488/Propidium Iodide (PI) kit (Invitrogen, California, USA) was employed. The INS-1 cells were divided into 4 

groups: control group; high glucose (30 mmol/l glucose) + high fat (400 μmol/l palmitic acid sodium) group; high glucose (30 mmol/l glucose) + high fat (400 μmol/l palmitic acid sodium) + oxymatrine (10 μmol/l) group; high glucose (30 mmol/l glucose) + high fat (400 μmol/l palmitic acid sodium) + oxymatrine (10 μmol/l) + stromatoxin-1 (100 nmol/l) group. After treatment with the corresponding drugs for 12 hr or 24 hr, the cells were collected, washed with PBS, and centrifuged (1000 r/min, 5 min). The supernatant was discarded. Subsequently, the cells were resuspended in binding buffer (1 × Annexin) to a density of 1×10^6^ cells/ml and stained with Alexa Fluor 488 annexin V/PI according to the manufacturer’s instructions. After incubation at room temperature for 15 min, the cells were detected by a FACS Calibur flow cytometer (BD Biosciences, USA). 

***Western blot experiments***

The protein levels were tested by Western blot. Anti-Bax antibody (ab32503), Anti-Bcl-2 antibody (ab196495), Anti-K_V_2.1 antibody (ab192761) were purchased from Abcam (Cambridge, UK); Anti-caspase-3 antibody (9662) was from Cell Signaling Technology (Boston, USA); β-actin antibody and corresponding secondary antibodies were obtained from Abcam (Cambridge, UK). The intensity of protein bands was determined using Image-Pro Plus 6.0.

***5-Ethynyl-2’-deoxyuridine (EdU) assay***

INS-1 cell proliferation was assessed by the 5-Ethynyl-2’-deoxyuridine (EdU) Cell Proliferation Detection Kit (Ribo Life Science, Suzhou, China) in accordance with the manufacturer’s instructions. In brief, 8 × 10^3^ cells/well were seeded in 96-well plates. INS-1 cells were divided into 4 groups: control group; high glucose (30 mmol/l glucose) + high fat (400 μmol/l palmitic acid sodium) group; high glucose (30 mmol/l glucose) + high fat (400 μmol/l palmitic acid sodium) + oxymatrine (10 μmol/l) group; and high glucose (30 mmol/l glucose) + high fat (400 μmol/l palmitic acid sodium) + oxymatrine (10 μmol/l) + stromatoxin-1 (100 nmol/l) group. After incubation for 24 hr, the cells were treated with EdU (50 μmol/l) for 2 hr. Then, the cells were fixed, discolored, and stained with Hoechst 33342. Photographs of cells were taken using a laser confocal microscope (LSM800, Carl Zeiss, Germany).

***Statistical analysis***

All data were presented as mean ± standard deviation (SD). Statistical significance was determined using analysis of variance (ANOVA) followed by Tukey’s test. *P*<0.05 was considered significant.

## Results

***Oxymatrine promotes glucose-stimulated insulin secretion in rat pancreatic islets***

**Figure 1.**
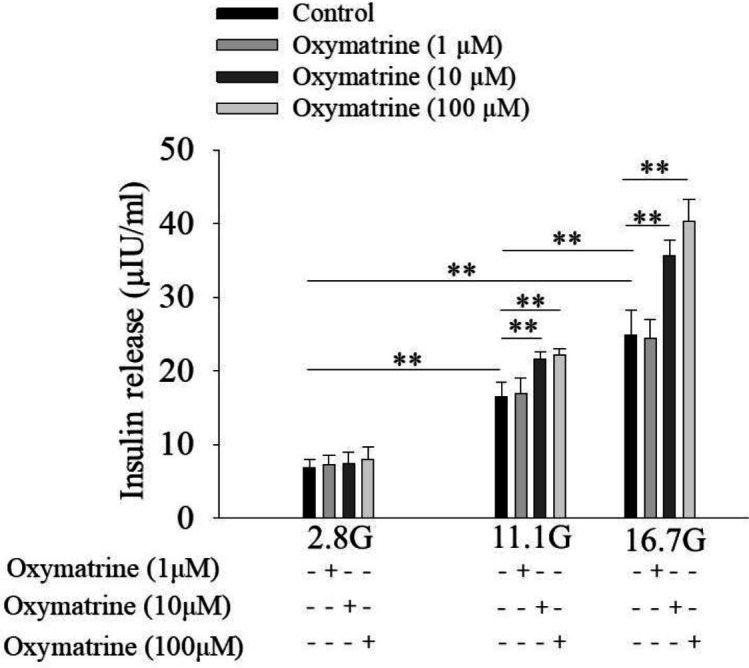
Oxymatrine promotes glucose-stimulated insulin secretion in rat pancreatic islets. Rat islets were incubated in Krebs-Ringer bicarbonate-HEPES buffer containing 2.8 mmol/l glucose (2.8 G), 11.1 mmol/l glucose (11.1 G), or 16.7 mmol/l glucose (16.7 G) in the absence or presence of oxymatrine as indicated. Each tube contains 6 islets, each group contains 7 tubes.**P<0.01

We tested the effects of different doses of oxymatrine (1 μmol/l, 10 μmol/l, and 100 μmol/l) on insulin secretion. As shown in Figure 1, oxymatrine (1 μmol/l, 10 μmol/l, and 100 μmol/l) had no effect on insulin secretion at low glucose level (2.8 mmol/l) (*P*>0.05 vs 2.8 G). In contrast, at high glucose levels (11.1 mmol/l, 16.7 mmol/l), oxymatrine (10 μmol/l and 100 μmol/l) promoted insulin secretion (*P*<0.01 vs 11.1 G, *P*<0.01 vs 16.7 G). Furthermore, 10 μmol/l oxymatrine increased insulin secretion (21.69 ± 0.92) by 31% under 11.1 mmol/l glucose conditions (16.52 ± 2.04) (*P*<0.01 vs 11.1 G) and significantly potentiated insulin secretion (35.62 ± 2.20) by approximately 44% at 16.7 G (24.81 ± 3.46) (*P*<0.01 vs 16.7 G).

***Oxymatrine inhibits K***_V_ ***channels in rat islet β cells***

**Figure 2.**
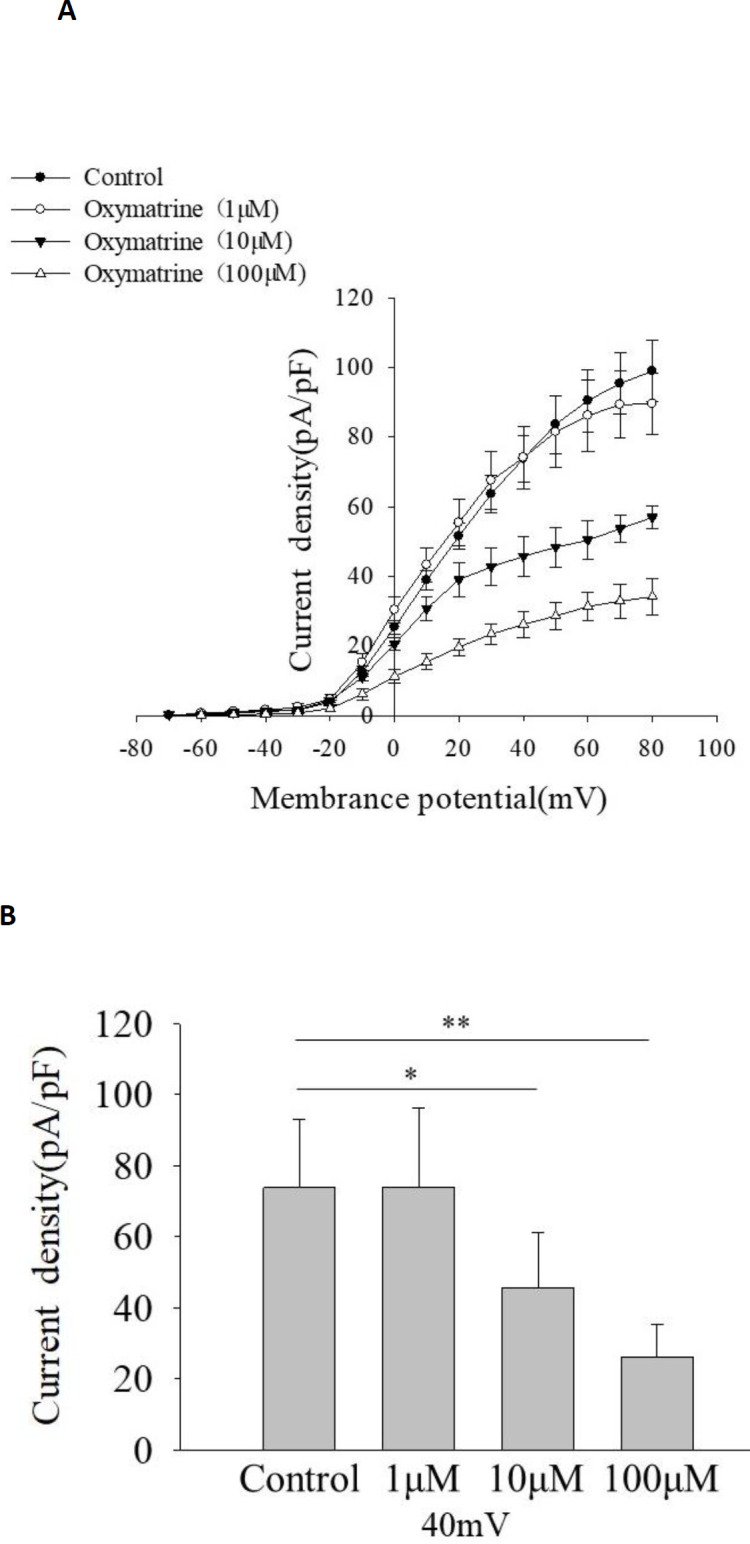
Oxymatrine inhibits kv channels in rat islet β cells. The cells were treated with or without oxymatrine (1 μmol/L,10 μmol/l, and 100 μmol/l). kv currents were recorded 10 min after different treatments from a holding potential of -70 mV to various test pulses (-70 to 80 mV) in 10 mV steps. (A) Current-voltage curves of Kv channel currents under different treatments. (B) Summary of the mean current density of Kv channel currents at 40 mV (each group represents different cells, n=6 β cells/group), *P<0.05, **P<0.01

To study if Kv channels are involved in oxymatrine-regulated glucose-stimulated insulin secretion, we detected K_V_ channel currents by the whole-cell voltage-clamp technique. All currents recorded were represented as current density [picoampere (pA) /picofarad (pF)]. Figure 2A shows that 10 μmol/l and 100 μmol/l oxymatrine inhibited K_V_ channel currents compared with Control. Oxymatrine (10 μmol/l) produced approximately 38% inhibition of K_V_ currents at 40 mV (45.73 ± 15.34 pA/pF for 10 μmol/l oxymatrine, 73.80 ± 19.23 pA/pF for control, *P*<0.05 vs Control, Figure 2B). 

***Oxymatrine increases the expression level of K***_V_ ***2.1 channel protein in INS-1 cells***

**Figure 3:**
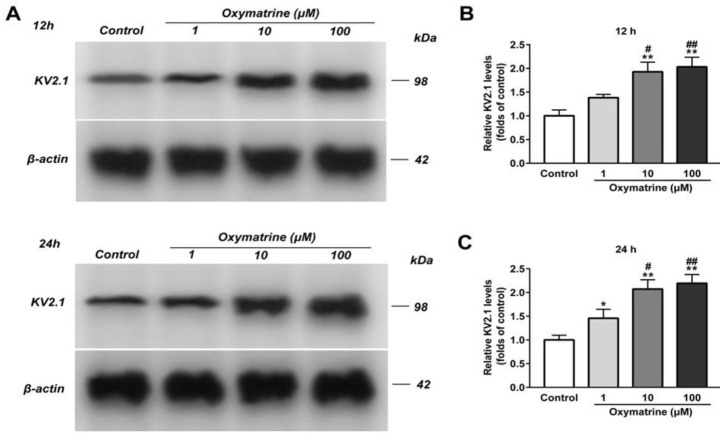
Oxymatrine increases the expression level of kv2.1 channel protein in INS-1 cells. The INS-1 cells were treated with or without oxymatrine (1 μmol/l,10 μmol/l, and 100 μmol/l). The expression level of kv2.1 channel protein was detected by Western blot assay, (A) Representative Western blot bands of kv2.1 channel protein were shown. (B) Analysis of the amount of kv2.1 channel protein (normalized to control). The experiments were repeated three times. Data represent an average of three experiments, 2×10^5^ cells/well. *P<0.05 vs Control, **P<0.01 vs Control, #*P*<0.05 vs Oxymatrine (1 μmol/l), ##*P*<0.01 vs Oxymatrine (1 μmol/l)

Compared with the control group, oxymatrine (1 μmol/l) increased the expression level of K_V_2.1 channel protein after INS-1 cells were incubated for 24 hr (Figures 3A, C, *P*<0.05 vs Control), but no effect at 12 hr (Figures 3A, B, *P*>0.05 vs Control). In contrast, oxymatrine (10 μmol/l,100 μmol/l) significantly up-regulated K_V_2.1 channel protein at 12 hr and 24 hr (Figures 3A-C, *P*<0.01 vs Control). But there was no distinct difference between the two groups (Figure 3A-C, *P*>0.05).

***Oxymatrine promotes cell viability in INS-1 cells under high glucose and high-fat conditions***

**Figure 4:**
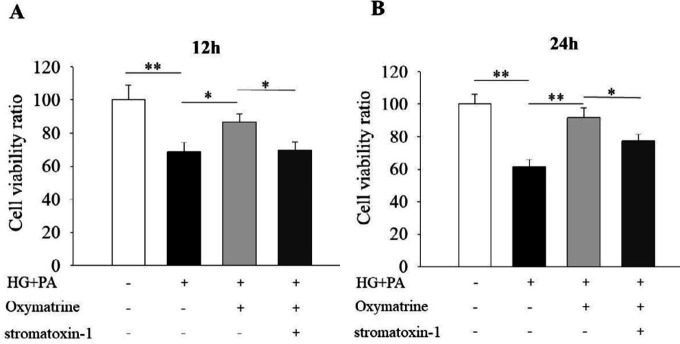
Oxymatrine promotes cell viability in INS-1 cells under high glucose and high-fat conditions. The INS-1 cells were treated with or without oxymatrine (10 μmol/l) for 12 hr and 24 hr under different conditions. Data are normalized to control. INS-1 cells were preincubated with stromatoxin-1 for 30 min before the addition of oxymatrine, high glucose, and palmitic acid sodium. Then, the INS-1 cells were treated together with all corresponding drugs for 12 or 24 hr. The experiments were repeated three times. Data represent an average of three experiments, 8×10^3^ cells/well. Control: not treated; HG: high glucose (30 mmol/l glucose); PA: palmitic acid sodium (400 μmol/l); stromatoxin-1 (a kv channel inhibitor, 100 nmol/l). *P<0.05, **P<0.01

To evaluate the effect of oxymatrine on INS-1 cell viability, CCK-8 experiments were used. The results indicated that the INS-1 cell viability was significantly reduced in the high glucose and high fat group (*P*<0.01 vs Control, Figure 4). After the cells were treated with oxymatrine for 12 and 24 hr, the survival rate was increased (*P*<0.05 vs HG + PA, Figure 4A;* P*<0.01 vs HG + PA, Figure 4B). The protective effect of oxymatrine was inhibited by applying a K_V_ channel inhibitor, stromatoxin-1 (*P*<0.05 vs HG + PA + oxymatrine, Figure 4).

***Oxymatrine increases the proliferation capacity of INS-1 cells in high glucose and high fat***

**Figure 5:**
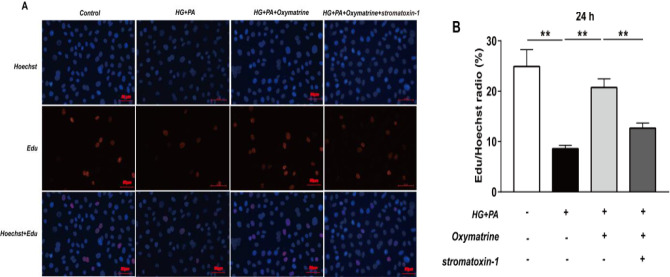
Oxymatrine increases the proliferation capacity of INS-1 cells in high glucose and high-fat. The proliferating nuclei were stained red with EdU and the nuclei of all cells were stained blue with Hoechst 33342. (A) Representative pictures from confocal microscopy were shown. (B) Analysis of the numbers of EdU positive and Hoechst 33342 positive cells. INS-1 cells were preincubated with stromatoxin-1 for 30 min before the addition of oxymatrine, high glucose, and palmitic acid sodium. Then, the INS-1 cells were treated together with all corresponding drugs for 24 hr. The experiments were repeated three times. Data represent an average of three experiments, 8×10^3^ cells/well. Control: not treated; HG: high glucose (30 mmol/l glucose); PA: palmitic acid sodium (400 μmol/l); stromatoxin-1 (100 nmol/l); 5-Ethynyl-2’-deoxyuridine: Edu, **P<0.01

To elucidate if oxymatrine affects the proliferation ability of INS-1 cells in high glucose and high fat, a 5-Ethynyl-2’-deoxyuridine (EdU) assay was performed. Compared with the control group (24.92% ± 3.35%), the cell proliferation ability was significantly decreased in the high glucose and high fat group (8.58% ± 0.67%) (*P*<0.01 vs Control, Figure 5). After the cells were incubated with oxymatrine for 24 hr, the proliferation ability was increased (20.73 ± 1.73%) (*P*<0.01 vs HG + PA, Figure 5), which could be reversed by using Stromatoxin-1 (*P*<0.01 vs HG + PA + oxymatrine, Figure 5).

***Oxymatrine reduces high glucose and high fat-induced INS-1 cell apoptosis***

**Figure 6:**
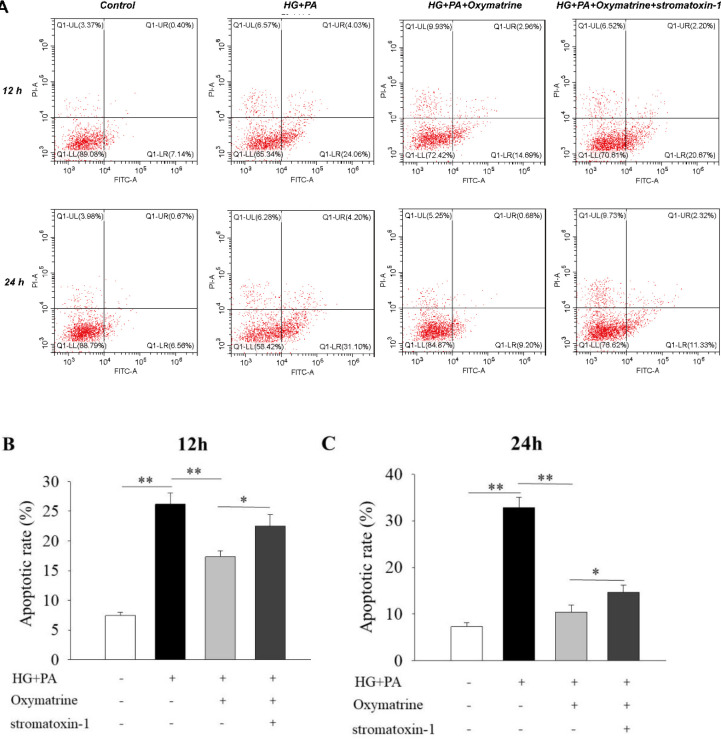
Oxymatrine reduces high glucose and high fat-induced INS-1 cell apoptosis. (A) Representative pictures from flowcytometery analysis were shown. (B) The apoptotic rate analysis of different groups. INS-1 cells were preincubated with stromatoxin-1 for 30 min before the addition of oxymatrine, high glucose, and palmitic acid sodium. Then, the INS-1 cells were treated together with all corresponding drugs for 12 or 24 hr. The experiments were repeated three times. Data represent an average of three experiments, 1×10^6^ cells/ml. Control: not treated; HG: high glucose (30 mmol/l glucose); PA: palmitic acid sodium (400 μmol/l); stromatoxin-1 (100 nmol/l); *P<0.05, **P<0.01

As shown in Figure 6, the apoptotic rate of INS-1 cells in high glucose and high-fat groups was obviously increased compared with the control group (*P*<0.01 vs Control, Figure 6). After the cells were treated with oxymatrine for 12 hr and 24 hr, the apoptotic rate was significantly reduced, reaching 17.30% ± 1.00% at 12 hr and 10.35% ± 1.52% at 24 hr (*P*<0.01 vs HG + PA, Figures 6 B, C). By employing Stromatoxin-1, the effect of oxymatrine on INS-1 cell apoptosis was suppressed (*P*<0.05 vs HG + PA + oxymatrine, Figures 6 B, C).

***Oxymatrine affects the protein expressions of Bax, Bcl-2, and Caspase-3 in INS-1 cells under high glucose and high-fat conditions***

**Figure 7:**
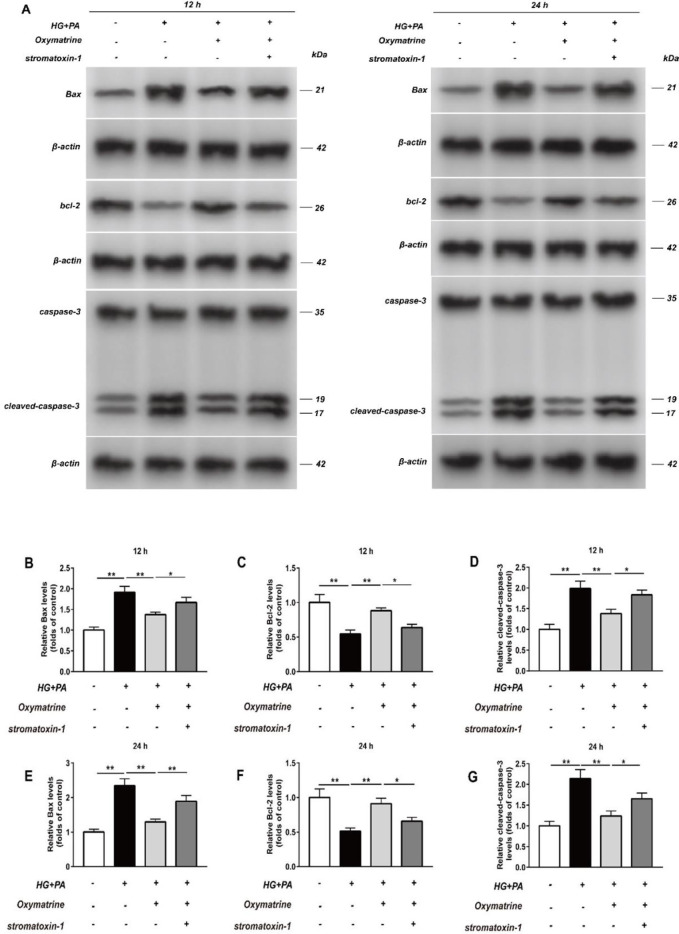
Oxymatrine affects the protein expressions of Bax, Bcl-2, and Caspase-3 in INS-1 cells under high glucose and high-fat conditions. The INS-1 cells were treated with or without oxymatrine (10 μmol/l) for 12 hr and 24 hr under different conditions. Data are normalized to Control. INS-1 cells were preincubated with stromatoxin-1 for 30 min before the addition of oxymatrine, high glucose, and palmitic acid sodium. Then, the INS-1 cells were treated together with all corresponding drugs for 12 or 24 hr. The experiments were repeated three times. Data represent an average of three experiments, 2×10^5^ cells/well. Control: not treated; HG: high glucose (30 mmol/l glucose); PA: palmitic acid sodium (400 μmol/l); stromatoxin-1 (100 nmol/l); (A) Representative Western blot bands of Bax, Bcl-2 and Caspase-3 were shown. (B, E) Analysis of the amount of Bax protein. (C, F) Analysis of the amount of Bcl-2 protein. (D, G) Analysis of the amount of Caspase-3 protein. *P<0.05, **P<0.01

As indicated, the protein expressions of Bax, Bcl-2, and Caspase-3 from different groups were detected by Western blot assay. In the high glucose and high fat group, the protein expressions of Bax and Caspase-3 were significantly increased (*P*<0.01 vs Control, Figures 7A, B, D, E, and G), but the Bcl-2 level was decreased (*P*<0.01 vs Control, Figures 7A, C, and F). As expected, after the cells were incubated with oxymatrine for 12 and 24 hr, Bax and Caspase-3 protein levels were reduced (*P*<0.01 vs HG + PA, Figures 7A, B, D, E, and G). At the same time, the Bcl-2 level was increased (*P*<0.01 vs HG + PA, Figures 7A, C, F). The effects could be reversed by adding Stromatoxin-1 (*P*<0.01 vs HG + PA + oxymatrine, Figure 7E; *P*<0.05 vs HG + PA + oxymatrine, Figures 7B, C, D, F, and G)

## Discussion

In the current study, we investigated the role of oxymatrine in rat islet cells. The results indicated that oxymatrine stimulated insulin secretion in isolated rat islets, which was similar to the previous report (22, 23). Furthermore, we found that oxymatrine-regulated insulin secretion depended on glucose concentrations. Because oxymatrine increased insulin secretion under high glucose conditions (11.1 mmol/l and 16.7 mmol/l) but had no effect at low glucose (2.8 mmol/l). We also studied the role of oxymatrine in INS-1 cells. Our data showed that oxymatrine promoted cell viability and proliferation, and suppressed cell apoptosis in INS-1 cells incubated with high glucose and high fat. Oxymatrine decreased the protein levels of Bax and Caspase-3, and increased Bcl-2 level, which suggests that these apoptosis-associated proteins are involved in oxymatrine-inhibited INS-1 cell apoptosis.

K_V_ channels are related to insulin secretion, cell proliferation, and apoptosis. The insulinotropic effects of Kv channel inhibition depend on glucose concentrations (9,32), which is in line with our secretion results. Therefore, we tested if K_V_ channels affected the effects of oxymatrine on isolated rat islets β cells and INS-1 cells. The results showed that oxymatrine suppressed the K_V_2.1 channel in rat islet β cells, but increased the expression level of K_V_2.1 protein in INS-1 cells. We have no immediate explanation for this discrepancy. It is likely explained by acute and chronic treatment of oxymatrine. Studies have found that the Kv2.1 channel may have acute and chronic manifestations in different conditions. There may be oxidation of Kv2.1 channels and reduction of currents immediately following oxidative damage (33, 34), followed by soluble NSF attachment receptor (SNARE)-dependent trafficking of Kv2.1 channels to the plasma membrane, resulting in the increase of K^+ ^current (35, 36). Similarly, oxymatrine also shows acute and chronic effects on K^+^ channels. A study recorded delayed rectifier K^+^ channel from clonal pheochromocytoma (PC12) cells with automatic patch-clamp. Each concentration of oxymatrine was added once to the cells and lasted at least 300 sec until currents reached equilibrium. The results show that oxymatrine decreases the peak amplitudes of delayed rectifier K^+^ currents in PC12 cells (29). Hu *et al.* also studied the acute effect of oxymatrine on the HEK293 cells that stably expressed the wild-type hERG gene. Oxymatrine at 30 °C inhibits hERG tail current in a concentration-dependent manner (cells were superfused continuously for about 10 min) (30). But a study indicates that the acute application of oxymatrine has no effect on probucol-induced hERG deficiency (data not shown). This demonstrates that the acute effects of oxymatrine may depend on the regulation of the kinetics of hERG channel gating (37). Zhang *et al.* investigated the effects of oxymatrine on the rapidly activating, delayed rectifier potassium channel (IKr) encoded by hERG. They show that the expression of the hERG protein and hERG currents are increased when hERG-human embryonic kidney (HEK 293) cells (a stably transfected HEK 293 cell line expressing high-level functional hERG) were cultured for 24 hr in the presence of oxymatrine (1 µmol/l) (38).

It seems that oxymatrine cannot be described as a Kv channel inhibitor or a Kv channel activator. Research proved that Ca^2+ ^influx through voltage-gated Ca^2+ ^channels contributes to β cell apoptosis in mouse primary β cells and in a pancreatic β cell line (39). Ca^2+^ has similar effects on pancreatic β cells from ob/ob mice treated with high concentrations of glucose (40). Activation of L-type Ca^2+^ channels is related to DNA fragmentation characteristic of apoptosis and the blockers of these channels suppress endonuclease activation (41). When K^+^ channels are activated, the cell membrane is hyperpolarized and Ca^2+^ is prevented from entering the cell through a voltage-dependent Ca^2+^ channel (3). At the same time, glucose-stimulated insulin secretion was compromised (9). 

On the other hand, cell shrinkage is an early prerequisite for apoptosis, and intracellular K^+^ loss is a major cause for cell shrinkage because K^+^ is the primary cation inside the cell determining intracellular osmolarity. Increased K^+^ efflux has been proven to be related to the early stage of apoptosis in many cell types (3, 42-44). As previously discussed by Chu *et al.*, increased expression of the Kv channel may be a protective mechanism in the β cell to inhibit insulin exhaustion and cell death but only for a period of time. The β cells treated with high glucose for more than 7 days showed irregular cell shape (45). 

Activation of K^+^ channels prevents Ca^2+^ from entering the cell, which inhibits cell proliferation (3). But in tumor cells, the effects of K^+^ channels-promoted cell proliferation are observed (46-49). One explanation is that membrane hyperpolarization increases the driving force for Ca^2+ ^entry into the cell and subsequently modulates cell proliferation (3). It is unclear whether the same mechanism operates in other cells. Therefore, the complicated relationships between oxymatrine and K^+ ^channels, and β cell growth remain to be further studied.

There is a limitation in our study. In the insulin secretion and electrophysiological experiments, we only observed the acute effects of oxymatrine. The chronic effects of oxymatrine on the electrical activity of the K_V_ channel and insulin secretion should be investigated. 

## Conclusion

Taken together, our results demonstrate that oxymatrine enhances insulin secretion, which is related to its influence on K_V_2.1 channels. In addition, oxymatrine also promotes cell viability and proliferation, and inhibits cell apoptosis in INS-1 cells under high glucose and high-fat conditions, which has a relationship with the K_V_2.1 channel. The double mechanism of oxymatrine on the K_V_2.1 channel needs to be further studied.
